# Navigation Patterns and Scent Marking: Underappreciated Contributors to Hippocampal and Entorhinal Spatial Representations?

**DOI:** 10.3389/fnbeh.2018.00098

**Published:** 2018-06-05

**Authors:** Mikhail A. Lebedev, Alexey Pimashkin, Alexei Ossadtchi

**Affiliations:** ^1^Department of Neurobiology, Duke University, Durham, NC, United States; ^2^Center for Bioelectric Interfaces of the Institute for Cognitive Neuroscience of the National Research University Higher School of Economics, Moscow, Russia; ^3^Laboratory of Neuroengineering, Center of Translational Technologies, Lobachevsky State University of Nizhni Novgorod, Nizhny Novgorod, Russia

**Keywords:** navigation behavior, hippocampal formation, grid cells, head direction cells, chicken or egg dilemma, scent marking, place cells

## Abstract

According to the currently prevailing theory, hippocampal formation constructs and maintains cognitive spatial maps. Most of the experimental evidence for this theory comes from the studies on navigation in laboratory rats and mice, typically male animals. While these animals exhibit a rich repertoire of behaviors associated with navigation, including locomotion, head movements, whisking, sniffing, raring and scent marking, the contribution of these behavioral patterns to the hippocampal spatially-selective activity has not been sufficiently studied. Instead, many publications have considered animal position in space as the major variable that affects the firing of hippocampal place cells and entorhinal grid cells. Here we argue that future work should focus on a more detailed examination of different behaviors exhibited during navigation to better understand the mechanism of spatial tuning in hippocampal neurons. As an inquiry in this direction, we have analyzed data from two datasets, shared online, containing recordings from rats navigating in square and round arenas. Our analyses revealed patchy navigation patterns, evident from the spatial maps of animal position, velocity and acceleration. Moreover, grid cells available in the datasets exhibited similar periodicity as the navigation parameters. These findings indicate that activity of grid cells could affect navigation parameters and/or vice versa. Additionally, we speculate that scent marks left by navigating animals could contribute to neuronal responses while rats and mice sniff their environment; the act of sniffing could modulate neuronal discharges even in virtual visual environments. Accordingly, we propose that future experiments should contain additional controls for navigation patterns, whisking, sniffing and maps composed of scent marks.

The mainstream theory of the hippocampal formation, recognized by the 2014 Nobel prize in Physiology or Medicine (Burgess, 2014), claims that this brain region constructs a cognitive map of space (O’Keefe and Nadel, 1978; Moser et al., 2008). John O’Keefe pioneered this idea in the 1970s. He advocated a neuro-ethological approach to rodent neurophysiology, where neuronal activity is examined during normal animal behavior, such as foraging (O’Keefe and Nadel, 1978). Using this approach, O’Keefe and Dostrovsky (1971) discovered place cells in the rat hippocampus that fired predominantly when a rat entered a particular spatial location. O’Keefe and Conway (1978) also demonstrated a strong relationship between the hippocampal place fields and environmental visual cues. Moser et al. (2008) commented on this development, “Early on, it became apparent that place fields are strongly influenced by distal sensory cues.” This key finding instigated a large number of studies on the generation of complex spatial maps by the hippocampal formation from multiple sensory inputs and motor information (McNaughton et al., 1996, 2006; Save et al., 2000; Chen et al., 2013; Zhang and Manahan-Vaughan, 2013). As this research progressed, simple explanations of hippocampal activity, like neuronal responses to odors (Vanderwolf, 1992, 2001), have been replaced by the interpretations in terms of hierarchical cortical processing (Moser and Moser, 2013).

A typical study that examines hippocampal place cells and/or entorhinal grid cells would consider neuronal activity as a function of animal position; the contribution of speed and head direction would be also analyzed. Taking as an example the studies on grid cells in the entorhinal cortex (Fyhn et al., 2004; Hafting et al., 2005), an open-field paradigm would be used. An experimenter would scatter pieces of food (chocolate crumbs or Froot Loops) to encourage animal exploration of the entire field. Since the animal apparently exhibits near-random trajectories, the navigation trajectories are presumed to be irregular and their structure is not thoroughly examined. Yet, this simplified description does not account for many behaviors exhibited by mice and rats navigating in novel and familiar environments (Thompson et al., 2018). Such navigation is typically patterned. For example, during navigation in an open-field arena, an animal would establish a home base, travel mostly along the walls, and only occasionally advance into the open field (Golani et al., 1993; Drai and Golani, 2001; Benjamini et al., 2010; Yaski et al., 2011; Thompson et al., 2018). Furthermore, when mice and rats navigate in their environment, they exhibit many distinct behaviors, such as locomotion (Parker and Clarke, 1990; Vásquez et al., 2002; Eilam et al., 2003), whisking (Berg and Kleinfeld, 2003; Leiser and Moxon, 2007; Mitchinson et al., 2007), sniffing (Welker, 1964; Clarke et al., 1970; Kepecs et al., 2005) and their combinations (Cao et al., 2012; Ranade et al., 2013; Fonio et al., 2015).

We hypothesized that the aforementioned factors—namely different behaviors accompanying navigation—could influence spatially-related neuronal responses that are described in the literature as neuronal spatial maps. Thus, the crystalline-like hexagonal patterns exhibited by grid cells in the entorhinal cortex (Fyhn et al., 2004; Hafting et al., 2005) could be related to certain behaviors that exhibit spatial periodicity. For example, an animal could move seemingly chaotically but in actuality “paint” a hexagonal grid, perhaps somewhat like swimming bacteria that build a hexagonal-patterned veil on sulfidic marine sediment (Cogan and Wolgemuth, 2005). In the case of a navigating rat or mouse, hexagonal-shaped neuronal patterns could be related to sniffing, head and body turns, locomotion onsets and offsets etc. — the behaviors that form the grid nodes. In support of our hypothesis, plots can be found in the existing literature that reveal patchy navigation patterns: Figure 8A in Wells et al. (2013), Figure 2A in Tuma et al. (2015), Figure 1 in Beynon and Hurst (2004), Figure 1 in Jamon (1994) and Figure 3 in Thompson et al. (2018). Structured navigation patterns are also visible in some of the figures shown in the grid-cell studies, but there they are usually difficult to read because of the cluttered presentation, with low-resolution images of the navigation trajectories and large dots that represent neuronal spikes being superimposed over the trajectories. (e.g., Figure 2A in Miao et al., 2017). Some studies of grid cells do not show any trajectories at all (e.g., Krupic et al., 2018).

To test our hypothesis, we examined the data taken from two shared datasets. The first dataset^1^ came from the study of Mizuseki et al. (2009a) and the second^2^ from the study of Hafting et al. (2005). We used position data from the first dataset and both position and neuronal data from the second dataset.

In these data, we found evidence of grid-like spatial patterns in the navigation parameters. Figure 1 shows our analysis of three experimental sessions from the Mizuseki et al. (2009a) dataset hc2: ec013.527 (Figures 1A,D,G,J), ec013.528 (Figures 1B,E,H,K), and ec013.755 (Figures 1C,F,I,L), all obtained from the same male rat foraging in a 180 cm by 180 cm square arena. The first two sessions were conducted on the same day, and the third one 11 days after. Upon visual inspection, rat trajectories (Figures 1A–C) are clearly patterned, with a prominent home base (bottom, middle) and patches of frequently visited places located near the walls and inside the arena. One can notice that the rat tended to repeat the same paths and did not visit certain places; some of these navigation patterns persisted during the day and even many days later. Additionally, the color-coded occupancy maps (Figures 1D–F) confirm the presence of frequently visited places in a grid-like arrangement. Next, we calculated vector fields for animal velocity (Figures 1G–I) and acceleration (not shown) by binning x and y dimensions into 200 bins, calculating average velocity and acceleration for each bin and smoothing bin values with a Gaussian spatial filter. Additionally, we calculated divergence (Figures 1G–I) and curl (not shown) for the vector fields. Divergence is scalar field generated from a vector field; it corresponds to the field’s source at every point. In relation with the animal’s behavior, divergence is negative when tracks converge (“sink”) and it is positive when the tracks diverge (“source”). The curl is a vector that characterizes rotation of a vector field. For a two-dimensional field, curl is perpendicular to the plane of the field, and its direction corresponds to the direction of rotation (clockwise or counterclockwise). In this case, curl can be represented by a scalar with the absolute value corresponding to rotation strength and the sign corresponding to rotation direction (positive for counterclockwise and negative for clockwise in our figures). With respect to the animal’s behavior, curl detects the points where the animal turns. Our analysis showed that the spatial distributions of divergence and curl exhibited periodicities, as confirmed by an autocorrelation analysis. (Figures 1J–L shows the autocorrelation for velocity divergence.) Overall, these patchy patterns resembled the spatial distributions previously reported for grid cells; although the periodicity was not as clear as for the best examples of grid cells, and no obvious hexagonal structure was visible.

**Figure 1:**
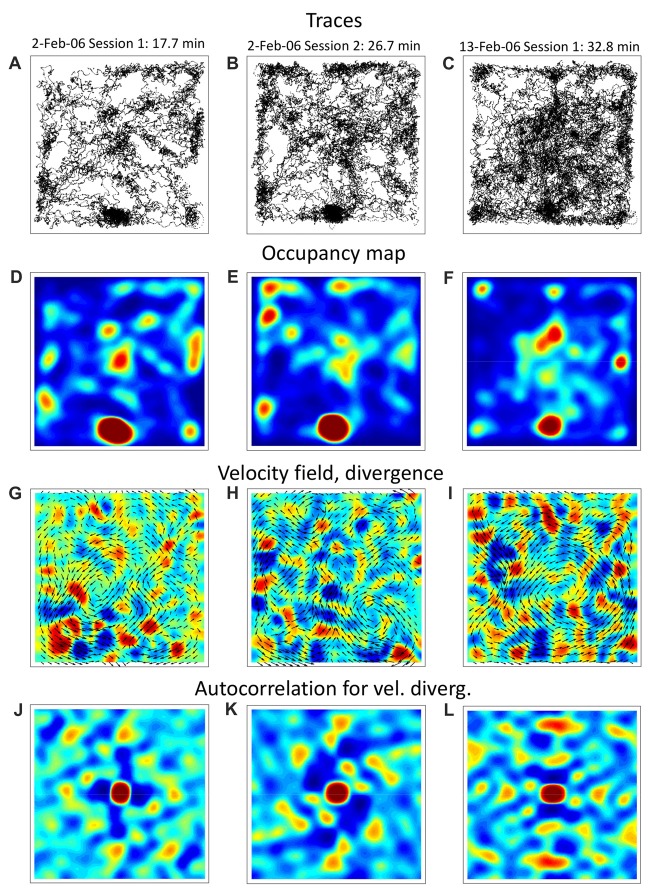
Navigation trajectories during open-field foraging in a square arena. Columns of panels (left, middle, right) correspond to different recording sessions for the same rat. **(A–C)** Trajectories from a rat foraging for Froot Loops in a square arena. **(D–F)** Occupancy map constructed from the trajectories. **(G–I)** Vector fields for navigation velocity plotted together with color-coded maps of vector-field divergence. **(J–L)** Autocorrelation for velocity divergence. Data was taken from the shared dataset: http://dx.doi.org/10.6080/K0Z60KZ9. Experimental sessions: hc2. Original study: Mizuseki et al. (2009a,b).

To test if spatial periodicities present in rat navigation patterns were correlated with the activity of grid cells, we analyzed data from the Hafting et al. (2005) dataset, where rat traces for a circular, 2 m in diameter, arena and examples of grid-cell activity were available. The results of this analysis are shown in Figure 2; data from two different rats (Figures 2A–R) are presented. For the navigation patterns, we analyzed navigation traces (Figures 2A,M), occupancy maps (Figures 2D,N), velocity field plotted together with its curl (Figure 2B), velocity field plotted with the divergence (Figure 2E), acceleration field plotted with the curl (Figure 2C) and acceleration field plotted with the divergence (Figures 2F,O). The curl and diverge, and to a less extent occupancy, showed spatial periodicity, as confirmed by autocorrelation analyses (not shown). Furthermore, Figure 2 shows an analysis of discharge patterns for two grid cells, one in each rat (Figures 2G–L,P–R). Both grid cells exhibited spatial periodicities, as evident from the spatial maps of their firing rate (Figures 2G,P) and autocorrelations (Figures 2J,Q). Moreover, crosscorrelation analysis revealed that the grid cells and the navigation patterns had matching spatial periodicities. This is evident from the crosscorrelation between the neuronal rate and velocity curl (Figure 2H), between the rate and acceleration curl (Figure 2I), between the rate and velocity divergence (Figure 2K), and between the rate and acceleration divergence (Figures 2L,R). Matching periodicity is particularly clear for the crosscorrelation with acceleration divergence, for both rats (and spatial filters could be adjusted to emphasize different harmonics in these plots; not shown). Although such a correlation analysis has caveats (e.g., a strong periodic pattern of a grid cell could dominate over a less regular navigation pattern), these results indicate that grid-cell responses may be related to the structure present in the navigation patterns.

**Figure 2:**
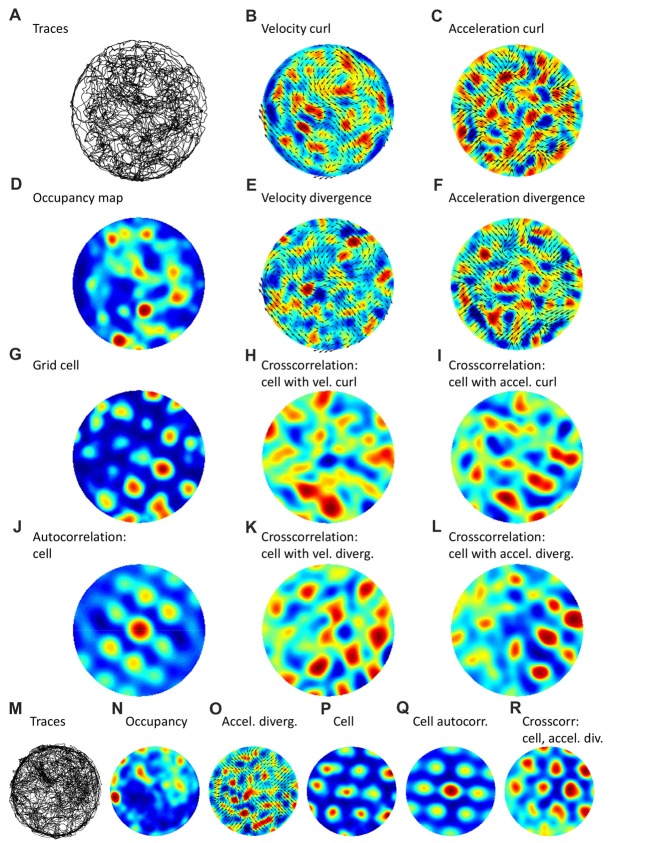
Navigation trajectories and grid-cell patterns during open-field foraging in a round arena. Panels **(A–L)** correspond to one rat, panels **(M–R)** to a different rat. **(A,M)** Trajectories from rats foraging for chocolate crumbs in a round arena. **(B)** Vector field for navigation velocity plotted together with a color-coded map of the field curl. **(C)** Vector field for acceleration, and its curl. **(D,N)** Occupancy maps for the trajectories shown in **(A,M)**, respectively. **(E)** Vector field for acceleration and its divergence. **(G,P)** Maps representing activity of grid cells. **(H)** Crosscorrelation between cell activity and velocity curl. **(I)** Crosscorrelation between cell activity and acceleration curl. **(K)** Crosscorrelation between cell activity and velocity divergence. **(L,R)** Crosscorrelation between cell activity and acceleration divergence. Data was taken from the shared dataset: https://www.ntnu.edu/kavli/research/grid-cell-data. Original study: Hafting et al. (2005).

The presence of roughly periodic patterns in the occupancy maps, as well as curl and divergence of the vector fields, suggests that the rats may have moved from node to node on a grid, the grid nodes corresponding to the points where they turned, stopped or accelerated. Indeed, high curl values correspond to turns, whereas high divergence value correspond to locations to which an animal comes from different directions (negative divergence) or where the animal takes off in various directions (positive divergence). These changes in kinematics could have been accompanied by additional behaviors, like sniffing, whisking, exploratory head movements, raring, etc.; each of these behaviors potentially reflected by the activity of grid cells. In support of this possibility, Monaco et al. (2014) have shown that head scanning behavior is involved in the expression of place cell activity, and Valerio and Taube (2012) have shown that head direction cell activity is modulated at home base locations.

The patchy patterns present in the rat navigation data may represent a behavioral correlate of the activity of grid cells. Indeed, if the entorhinal cortex generates a grid-like activity pattern, this neuronal activity could induce a behavior with a similar spatial structure. On the other hand, this argumentation runs into a “chicken or the egg dilemma”: could the spatially patterned behavior be the cause of the entorhinal grid and not vice versa? For example, rats may have sniffed near the grid nodes, causing the entorhinal neurons to fire. Food consumption is another candidate for the “chicken” (or “egg”, depending on the interpretation), so it would be of interest to measure the spatial distribution of chocolate crumbs as a function of time to determine the eating/foraging strategy adopted by the animal. It should be also mentioned that chewing causes mechanical artifacts that could interfere with the recordings of neuronal spikes.

Scent marks left by a navigating animal is another possible contributor to the grid-cell patterns (Lebedev and Ossadtchi, 2018). Indeed, as the animals move across the arena they may leave scent traces (Desjardins et al., 1973; Beynon and Hurst, 2004), and the map composed of scents eventually may start looking very much like the occupancy maps shown in Figures 1D–F, 2D–N. Accordingly, processing of the rat’s own odors may contribute to the periodic neuronal patterns. Curiously, when Hafting et al. (2005) placed their rats in total darkness for 30 min after an initial 10-min period with lights on, entorhinal grid patterns were little changed. This result suggests that the animals possibly utilized their own scent marks left inside the arena and near the walls. Alternatively, they may have performed path integration based on the body motion signals; however, in this case one would expect the neuronal spatial representation to drift because of the errors in path integration accumulated during the 30-min session.

In the wild, rats, mice and many other animals, exhibit territorial behaviors (Crowcroft, 1955; Mackintosh, 1970; Adams et al., 1994), including marking their territory with complex scent signals incorporating urinary proteins (Ralls, 1971; Alberts, 1992; Gosling and Roberts, 2001a,b; Beynon and Hurst, 2004; Hurst and Beynon, 2004). Male animals advertise their success with scent marks to gain competitive advantages and attract females (Roberts et al., 2010, 2012; Thonhauser et al., 2013). Moreover, rats and mice can utilize environmental odor cues to guide their navigation (Lavenex and Schenk, 1998; Wallace et al., 2002; Porter et al., 2005; Khan et al., 2012), although visual landmarks dominate over odor cues in certain cases (Small, 1901; Olton and Collison, 1979; Lavenex and Schenk, 1996; Maaswinkel and Whishaw, 1999).

Historically, Ramon Cajal initially believed that hippocampus was an olfactory area (DeFelipe and Jones, 1988; Vanderwolf, 2001). This theory was abandoned after Brodal (1947) reasoned that hippocampal formation was unlikely to have a role in olfaction because it did not appear to receive olfactory inputs, and hippocampal lesions had little effect on the olfactory conditioned behaviors. Brodal’s arguments turned out to be wrong: hippocampal formation does receive olfactory inputs (Krettek and Price, 1977; Luskin and Price, 1983; Room et al., 1984; Schwerdtfeger et al., 1990), and responds to odors electrophysiologically (Wilson and Steward, 1978; Vanderwolf, 1992; Biella and de Curtis, 2000; Insausti et al., 2002). More recently, several studies addressed the specific role of olfaction in the formation of hippocampal spatial maps (Goodridge et al., 1998; Save et al., 2000; Anderson and Jeffery, 2003; Zhang and Manahan-Vaughan, 2013). Jacobs (2012) suggested that navigation is the primary function of olfaction (as opposed to discriminating odors), and olfactory structures provide a scaffold for visually-guided navigation. Consistent with this idea, navigation is severely impaired in rats with olfactory bulbectomy, even when visual cues are available (van Rijzingen et al., 1995).

In addition to processing of odors, the act of sniffing itself can modulate hippocampal activity. Thus, O’Keefe’s has described “misplace units” in the hippocampus that were activated by sniffing when the rat encountered locations from which a familiar object was removed or where a new object was placed (O’Keefe, 1976). Additionally, olfactory system can be activated by the act of sniffing even in the absence of odors (Adrian, 1942; Macrides and Chorover, 1972).

Given this previous work, a control for the presence of scent marks could be useful for the studies of neuronal spatial properties. Scent marks could be revealed by illumination of the arena with ultraviolet light (Desjardins et al., 1973) or testing the floor for the presence of urinary protein using a polyclonal antibody (Beynon and Hurst, 2004). Matching the navigation traces and other behaviors to scent marks and comparing these landmarks with neuronal discharge rates could help to determine the contribution of odors to periodic spatial behaviors. The labs working with mice and rats usually clean their behavioral arenas in between the recording session, but this could be insufficient because 5–10 min may be enough for the animals to label their environment with scent marks. Thus, additional experimental controls for scent marking appear to be important for better understanding of navigation behaviors and neuronal responses associated with them.

Recently, place cells and grid cells have been reported in rats and mice navigating in virtual visual environments (Harvey et al., 2009; Dombeck et al., 2010; Chen et al., 2013; Domnisoru et al., 2013; Ravassard et al., 2013; Aronov and Tank, 2014; Aghajan et al., 2015). One of the goals of such virtual environments was to minimize the contribution of olfaction to the activity of neurons being studied. We speculate that even these virtual navigation traces may be found to contain periodicities, when analyzed. In support of this speculation, Figure 4A in Aronov and Tank (2014) and Figures 2A–C in Domnisoru et al. (2013) show non-uniform and possibly periodic occupancy maps. Curiously, the animals in these studies continued to sniff the treadmill although being immersed in the virtual world, as evident from Movie S1 of Aronov and Tank (2014), so the act of sniffing may have contributed to the observed neuronal responses.

In addition to contributing to a better understanding of grid cells, a thorough analysis of navigation patterns and navigation-associated behaviors could shed light on the mechanisms of spatial tuning in the other types of neurons, for example head-direction cells (Taube et al., 1990a,b), border cell (Solstad et al., 2008) and entorhinal cells with patchy responses lacking hexagonal structure. If kinematic parameters of navigation are different in different places of the arena, neuronal modulations to position, velocity and borders are all confounded; and different classes of neurons could be confused with each other unless the contribution of all navigation parameters is assessed.

While our manuscript was under review, Banino et al. (2018) published an article where they applied a long short-term memory (LSTM) recurrent network to generate place and head-direction activity (all in simulated neurons) from the simulated translational and rotational velocities taken from the model of rat navigation proposed by Raudies and Hasselmo (2012). The simulated rat navigated in a square arena. Under certain training conditions, units in LSTM linear layer (i.e., the layer that performed a linear transformation of the input velocities) exhibited hexagonal spatial patterns that resembled those of entorhinal grid cells. While the exact source of this emergent property of the network is difficult to identify, it is possible that the boundary conditions of the model resulted in spatial and temporal periodicities in the simulated rat trajectories and/or discharge patterns of place cells and head-direction cells. Raudies and Hasselmo required that the simulated rat started traveling along the wall after getting close to it; this requirement introduced a peculiar navigation pattern that resembled the behavior of a real rat. Given this peculiar behavior, a spatial pattern was introduced to the simulated activity of head-direction cells as they responded in a predetermined fashion when the simulated rat was near the wall. These possibilities could be examined in the future to better understand the origin of hexagonal patterns in the Banino et al. modeling results. Additionally, it would be of interest to test the same LSTM on real animal trajectories; the task could be as simple as predicting position from the instantaneous velocity and acceleration (both translational and rotational components), and head direction, if available in the recordings. (It is noteworthy that because of a non-uniform distribution of these variables across the arena, predictions of position can be performed even without integration over time, as evident from our initial assessment).

In conclusion, our analyses of the existing data indicate that rats foraging for scattered food exhibit spatially patterned navigation patterns that contain periodicities resembling the spatially-dependent responses of the entorhinal grid cells. Given this result, a detailed analysis of the relationship between the navigation patterns and neuronal activity could be helpful to achieve better understanding of the encoding properties of hippocampal and entorhinal neurons. Even if it turns out that spatial properties of behaviors, such as navigation, whisking, sniffing and scent marking, are quite different from the hippocampal spatial maps, such a negative result would still improve our understanding of how neuronal patterns affect behaviors and vice versa. Previously, Thompson et al. (2018) reached a similar conclusion.

## Ethics Statement

The manuscript contains an analysis of shared data: http://dx.doi.org/10.6080/K0Z60KZ9. Experimental sessions: hc2. Original study: Mizuseki et al. (2009b). https://www.ntnu.edu/kavli/research/grid-cell-data. Original study: Hafting et al. (2005). The original studies were approved by the institutional ethical committee.

## Author Contributions

ML, AP and AO contributed equally to the development of these ideas and data analyses.

## Conflict of Interest Statement

The authors declare that the research was conducted in the absence of any commercial or financial relationships that could be construed as a potential conflict of interest.

## Footnotes

^1^http://dx.doi.org/10.6080/K0Z60KZ9

^2^https://www.ntnu.edu/kavli/research/grid-cell-data
